# Aboveground Herbivory Shapes the Biomass Distribution and Flux of Soil Invertebrates

**DOI:** 10.1371/journal.pone.0003573

**Published:** 2008-10-31

**Authors:** Christian Mulder, Henri A. Den Hollander, A. Jan Hendriks

**Affiliations:** 1 Department of Ecology, National Institute for Public Health and the Environment, RIVM-LER, Bilthoven, The Netherlands; 2 Department of Environmental Science, Institute for Water and Wetland Research, Radboud University, Nijmegen, The Netherlands; Centre National de la Recherche Scientifique, France

## Abstract

**Background:**

Living soil invertebrates provide a universal currency for quality that integrates physical and chemical variables with biogeography as the invertebrates reflect their habitat and most ecological changes occurring therein. The specific goal was the identification of “reference” states for soil sustainability and ecosystem functioning in grazed *vs.* ungrazed sites.

**Methodology/Principal Findings:**

Bacterial cells were counted by fluorescent staining and combined direct microscopy and automatic image analysis; invertebrates (nematodes, mites, insects, oligochaetes) were sampled and their body size measured individually to allow allometric scaling. Numerical allometry analyses food webs by a direct comparison of weight averages of components and thus might characterize the detrital soil food webs of our 135 sites regardless of taxonomy. Sharp differences in the frequency distributions are shown. Overall higher biomasses of invertebrates occur in grasslands, and all larger soil organisms differed remarkably.

**Conclusions/Significance:**

Strong statistical evidence supports a hypothesis explaining from an allometric perspective how the faunal biomass distribution and the energetic flux are affected by livestock, nutrient availability and land use. Our aim is to propose faunal biomass flux and biomass distribution as quantitative descriptors of soil community composition and function, and to illustrate the application of these allometric indicators to soil systems.

## Introduction

The faunal biomass distribution related to the body sizes in different biota has recently become one major question for both the applied as the theoretical ecologists. Soil invertebrates have been thoroughly investigated during the last two decades. Soil communities are as complex as the inhabitants are numerous, making quantitative analyses of belowground invertebrates rare. Increased land use results in rapid decline of soil organic matter due to reduced input rates and decreased physical protection to decomposition by cropping and tillage. Animals linked to either labile or recalcitrant substrates support the complementarity action of “energy transfer agents” (nematodes and enchytraeids) or “habitat engineers” (earthworms), and controversy exists over whether soil invertebrates control (‘bottom–up’) aboveground primary productivity, or whether belowground changes in soil invertebrates follow (‘top–down’) changes in agroecosystems [1]–[6]. Such opposite, controversial trends also contributed to several other relevant questions [7]. Sutherland and co-authors identified 100 ecological questions of highest relevance, like the effects on biodiversity of farming systems such as organic, conventional, and integrated farm management (their question #9), the effects on soil functions of agricultural activities and practices (their #11) and “*the ecological consequences of changes in upland grazing regimes for biodiversity and soil ecology*” (their #12). Our paper will address this last question. Patterns of soil organisms, in fact, are supposed to provide one fine-tuned assessment of ecological processes occurring in belowground biota under different upland grazing regimes. Allometry provides fine tools to characterize networks, including mass balance and energy flux, by a direct comparison between differently-sized soil invertebrates with species-specific adult weight averages [8]. We document 135 soil communities to investigate faunal biomass distributions, food-web statistics and energy fluxes.

## Methods

### Soils

In this study, 55 ungrazed locations (19 Scots pine forests –traditional agroforestry– and 36 arable fields: intercropping, 14 fields, multicropping, 20 fields, and abandoned meadows, 2 old fields) and 80 grazed locations (21 organic grasslands, 19 dairy farms under conventional management and 40 (semi) intensively-managed farms) were sampled (Table 1). Farms constitute thus the basic sampling units for grazed agroecosystems and were grouped as previously described [5]: 21 certified organic grasslands (including mixed and bio-dynamic regimes), using compost/farmyard manure and no biocides, averaging 60 ha and 1.7 livestock units; 19 conventional farms, using mineral fertilisers, with a much smaller amount of farmyard manure, averaging 45 ha and 2.4 livestock units; 20 semi-intensive farms, using both organic and mineral fertilisers, averaging 25 ha and 3.0 livestock units; and 20 intensive farms, using biocides and fertilisers, averaging 20 ha and 5.1 livestock units. The livestock density was measured in terms of animal units, one unit corresponding to 41 kg P ha^−1^ excreted over one year (Table 1). Soils samples were analyzed in triplicate.

**Table 1:** Averages±standard deviation per agroecosystem type.

	Grass cover (%)	P-manure (kg ha^−1^ yr^−1^)	Soil C ∶ P ratio (mass units)
**GRAZED**			
organic grasslands	85.8±15.3	63.5±19.8	20.9±15.6
conventional farms	77.7±16.5	99.3±30.0	27.3±14.4
semi-intensive farms	73.8±23.2	124.3±33.1	14.0±4.8
intensive farms	76.0±21.7	266.7±148.8	11.5±3.7
**UNGRAZED**			
forests	2.6±11.5	0	472.4±347.4
fields	14.1±29.0	0	22.4±15.2

These types are characteristic for ∼70% of the Dutch rural landscape [42].

### Microflora and Microfauna

Microbiological samples were collected from the same soil samples as for nematodes and stored at a temperature of 12°C and 50% water holding capacity. Bacterial cells were counted in soil smears by fluorescent staining (5-(4, 6-dichlorotriazin-2-yl)-aminofluorescein (DTAF)) and direct co-focal laser scanning microscopy coupled to a fully automatic image analysis system [9]. Nematodes were extracted from 100 g soil using elutriation, sieving and cottonwool extraction [10], [11]. All individuals within two clean 10 ml water suspensions were screened, counted with a stereomicroscope and fixed in 4% formaldehyde. Per sample, at least 150 individuals were identified at genus level by light microscopy (400–600×) and assigned to feeding habits [12]. The length and width of 2186 nematodes was measured to estimate their individual fresh weight according to the volumetric method of Andrássy [13]. The estimated fresh weight was recalculated to dry weight according to a dry weight percentage of 0.20 [14], [15]. About 80% of the soil nematodes were identified to genus, the rest to family (Table S1).

### Mesofauna and Macrofauna

Microarthropods were collected in a randomized block design and their four-fold cores (diameter 5.8 cm×5 cm) were kept separate until behavioural extraction using the Tullgren high-gradient canister method with a low wattage bulb. The extraction from the core samples occurred within 15 days with the temperature gradient stepwise increasing from 20 to 60°C. Sampled microarthropods were observed and measured at a magnification of 200–1,000× with a light microscope and assigned to feeding guilds according to their specific enzymatic activity [16], [17]. Three carbohydrases have been measured: cellulase, chitinase, and trehalase. All these enzyme activities depend on the resources consumed prior to sampling [10], [11], [17], [18]. Enchytraeids were sampled using six-fold cores (diameter 5.8 cm×15 cm, 6 rings of 2.5 cm height each), extracted using wet funnel extraction, identified, measured and counted. Lumbricids were recovered manually, identified, weighted and counted.

Extracted microarthropods were divided in body-size classes (body length) to calculate the corresponding dry weight. Enchytraeids and lumbricids were measured individually to determine the specific average body size. From these body-size values, dry body-mass values were computed by volumetric relationships and assigned to each taxon recovered from any of the 135 sites. Of all the investigated taxa (146 mites, 41 collembolans, 12 enchytraeids and 9 lumbricids), more than 80% of the microarthropods and all the adult oligochaetes were identified to genus; the rest to family (Table S1). Merging at genus level did not introduce statistical biases: mean weight and standard deviation showed similar patterns for prey and predators [19]–[21].

### Networks

Soil community structure was described using food-web data with *M* (dry body mass in µg), *N* (animals/m^2^), and *B* (dry biomass in µg/m^2^, i.e. log(*B*) = log(*N*)+log(*M*)). A guild-lumped web was established for each site by taking the sub-predation-matrix determined by the trophic guilds that were present (binary matrix published online in Mulder et al. [11], their sub-predation-worksheet). For each site, this procedure gave log(*N*), log(*M*), and log(*B*) data attached to each node. The complete linear allometric model log(*B*) = *a*_1_ log(*M*)+*b*_1_ was fitted to these 135 sites separately (confidence interval 99%) and along the binned log(*M*) averages, the lumped log(*B*) for all samples taxa is plotted at the middle of that size class. Binned and lumped log(*B*) with zero observations are excluded, because log(0) is undefined. All statistical analyses were performed on SAS, version 9.1.3 Service Pack 3.

The ratio of production (*P*) at one trophic level (*i*) to the next trophic level *j* is a function of the proportion of the consumed resource *C_j_* and the conversion efficiency [22]–[24]. Soil organisms have been pooled into body-mass bins using the formula
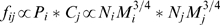

[24], [25], where *N* is the specific abundance (per square meter) and *M* is the specific adult body-mass average (µg dry weight elemental content across all life stages). Possible consumer–resource links were postulated; only pure-substrate ingestion by occurring lumbricids was not taken into account (detritus not quantifiable). These trophic links were defined according to Reuman and Cohen [26], where the *length l* of a link from the faunal prey (or the bacterial resource) *r* to the predator (consumer) *c* is:
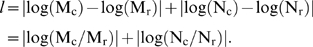
The presence or absence, but not the quantitative extent, of consumer–resource links was established using additional information from the literature, and summarized in the 5-digit codes shown in Table S1. We took in particular the mean *l*, the standard deviation of *l*, and the number of trophic links within our different agroecosystems into account. The *angle* α of any trophic link [26] was kept as the order of magnitude of the body-mass ratio between consumer and resource over the order of magnitude of the ratio between consumer and resource population densities, being:
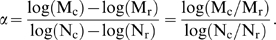
In order to understand our results, it is instructive to inspect the underlying distribution of nodes in an allometric (*N*,*M*) plane [11], [20], [21]; please note that if the two axes are inverted, the opposite holds. Each trophic link that has a slope equal to −1 (−45°) has consumer and resource of equal biomass *B*, a characteristic trait of steady-state systems. If α<−1 (steeper link), then the biomass of the consumer exceeds that of the resource; if α>−1 (shallower link), then the biomass of the resource exceeds that of the consumer. It also implies that a connection from a smaller taxon to a larger taxon, both of equal numerical abundance *N*, exhibits an angle of −90° (vertical trophic link), and a connection from a more (less) abundant resource to a less (more) abundant consumer, both of the same body mass *M*, exhibits an angle of 0° (horizontal trophic link). For instance, taking the bacterial-feeding nematodes into account, a slope more (or less) negative than −1 indicates that the microbial grazer has greater (or smaller) *biomass*, respectively, than the bacterial resource itself, assuming that the consumer is above and to the left of the resource as in [11]. However, in the case of prey-predator links, this assumption is not entirely true: at least one fifth of the faunal trophic links shows animals preying on invertebrates with the same body mass or an up to 4 orders of magnitude larger one. Cannibalism is widespread and omnivory is dominant.

## Results

### Biomass Spectra

**Figure 1:**
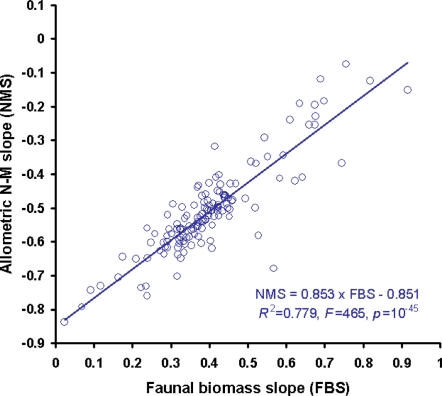
Linear binning and classic allometry are closely correlated. The arbitrary class interval of the log(*M*) bins for our 135 real webs is 0.2 with constant linear width. In contrast to the simulation results of White et al. [43], who generated power-law distributed random numbers using inverse transformation for the Pareto distribution [8], [43], the linear binning performed very well in our empirical study.

The allometric analyses showed that log(*N*), log(*M*), and log(*B*) are strictly correlated in our soil systems, as theoretically expected from lakes [8], [27]. The allometric size-abundance slopes (NMS, i.e. Numerical abundance as function of dry Mass averages) were always negative, whereas the faunal biomass–size slopes (FBS) were always positive in the investigated body-size range of our 135 soil systems. Merging the classic allometric formula log(*N*) = *a*×log(*M*)+*b* [25], [27] with log(*B*) = log(*M*)+log(*N*), we obtain log(*B*) = log(*M*)+*a*×log(*M*)+*b* = (1+*a*)×log(*M*)+*b*. Thus, both allometric slopes, NMS and FBS, are closely correlated (Figure 1, *R* ^2^ = 74%, *p*<10^−43^).

**Figure 2:**
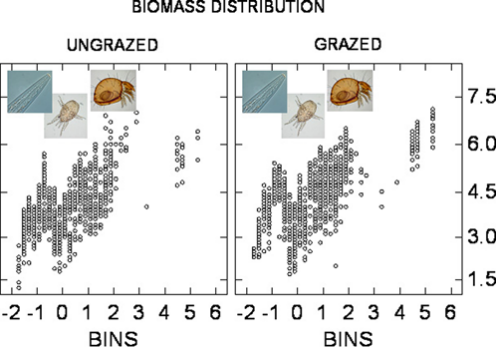
Distributions of log-transformed faunal biomass (ordinate) along a body size gradient (abscissa) for ungrazed and grazed agroecosystems. After lumping, grazed sites have but a higher biomass contribution of bacterial-feeding nematodes and a lower biomass contribution of hyphal-feeding enchytraeids than ungrazed sites. This structural compensation has at least two main consequences, one for the microbial consumption (microfauna grazing on bacteria, mesofauna browsing fungi) and the other for the soil aggregation and humification by larger arthropods. The peak in the biomass around 0.5 log(*M*) reflects the activity of gamasid mites (*Lysigamasus*, *Protodinychus*, *Uropoda* etc.) and predatory nematodes such as *Aporcelaimellus*. Some typical genera are shown: from left to right, *Chiloplacus*, a bacterial-feeding nematode highly tolerant for grazing pressure and land-use intensity, the predatory mite *Alliphis* and the microphytophagous *Rhysotritia* [10].

Figure 2 shows a striking multimodality in the biomass spectra for both the “grazed” and “ungrazed” meta-categories. Most fluctuations occurred within the microfauna (nematodes) and mesofauna (mites, collembolans, and enchytraeids). Soil faunal taxa exhibited a variety of relationships between biomass and binned body size within the investigated agroecosystems (Table 2). Faunal biomass–size slopes (FBS) ranged from a_1_ = 0.02–0.64 (arable fields), a_1_ = 0.07–0.53 (organic grasslands), a_1_ = 0.25–0.52 (semi-intensive farms), a_1_ = 0.26–0.45 (conventional farms), a_1_ = 0.29–0.69 (intensive farms), and a_1_ = 0.41–0.92 (forested sites). Intercepts of faunal biomass relationships ranged from b_1_ = 3.56–4.33 (arable fields), b_1_ = 3.60–4.41 (forested sites), b_1_ = 3.62–4.31 (intensive farms), b_1_ = 3.75–4.34 (organic grasslands), b_1_ = 3.81–4.43 (semi-intensive farms), and b_1_ = 3.90–4.53 (conventional farms). The most pronounced increase in the FBS occurred in forests, despite their lowest intercepts (Table 2). Our coefficients tend to decrease with the width of the body-size range covered by the linear regressions. Size bins seem to influence the resulting power functions, as previously reported by Siemann et al. [28]. Our faunal spectra tend to show a fluctuating increase in biomass with body size up to a peak near the largest weight-bins comparable to those of Duplisea and Drgas [29]. The latter implies that the micro– and mesofaunal biomass clump in grazed grasslands (10^4.16^ = 14,484 µg) is about two-fold that in ungrazed sites (10^3.90^ = 7,951 µg): less disturbance like grazing, trampling, manuring and tillage leads to lower intercepts of the biomass–body-size distribution (Pearson's Correlation equal to 0.227, *p* = 0.0059). On the other hand, soil nutrients seem to enhance the slope of the faunal biomass–body-size distribution (Table 2). That is, in P-enriched, intensively-managed soils, the biomass totals of the occurring larger soil animals tend to be greater relative to the biomass totals of the smaller animals than in infertile forests. In other words, the lack of nutrients in forests kills off or diminishes the relative abundance of large compared to small animals.

**Table 2:** Statistics of the soil faunal biomass distribution: linear regression slope, intercept, significance, food-web nodes and links with averages±standard deviation.

	Slope a_1_	Intercept b_1_	Significance *R* ^2^	Nodes (taxa)	Links #
organic grasslands	0.37±0.10	4.18±0.14	0.41±0.18	53±7	907±302
conventional farms	0.37±0.06	4.21±0.17	0.44±0.12	45±5	679±232
semi-intensive farms	0.38±0.06	4.16±0.15	0.43±0.14	46±8	804±296
intensive farms	0.41±0.09	4.10±0.16	0.54±0.12	62±7	1339±368
forests	0.63±0.13	3.89±0.19	0.61±0.13	77±9	1737±460
fields	0.32±0.12	3.92±0.19	0.27±0.16	50±10	744±455

Although forests belong to the only agroecosystem with a rather small width of the body-size range, the mean coefficient and significance of their biomass–size linear regressions are by far the highest. Not one of the log-log-scaled regression lines of biomass on binned body-mass was weak. Besides for forests, biodiversity at genus level was statistically undistinguishable between open-canopy ecosystems.

**Figure 3:**
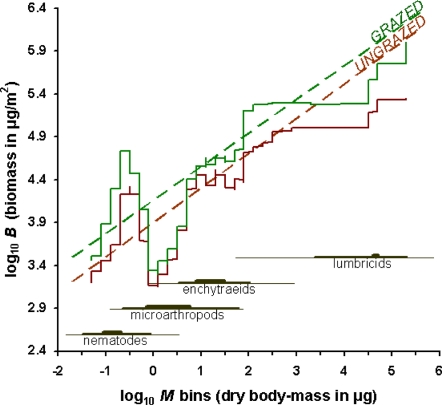
Repulsed frequency distribution of the faunal biomass (the aforementioned compensation between phyla is further supported by the parallel regression slopes and by the moving averages). With the chosen log(*M*) interval 86%±22 SD of the bins between −2 and 2 (microfauna and mesofauna) and 96%±7.5 SD of the bins between −1.6 and 0 (microfauna) are filled. All log(*M*) ranges are provided at the bottom (thin lines, min–max, medium lines, 5–95 percentile, thick lines, quartiles).

Although it is known from literature that the total biomass of above-ground and below-ground invertebrates in grasslands is much higher than in other ecosystems [30], [31], we are not aware of examples of *faunal biomass distribution* in soil systems. To address further the effects of macroherbivory on the soil system, we merged the biomass values for individually-binned size-classes together into Figure 3. The so-obtained coefficients of these two meta-FBS took both statistically indistinguishable values for either grazed or ungrazed systems (0.3917±0.0110 SE and 0.4042±0.0178 SE, respectively), in contrast to the vertical intercepts (4.1623±0.0208 SE and 3.9004±0.0248 SE, respectively). On the other hand, lumping these soil webs together made bimodal patterns detectable: according to the two moving averages, the microfauna clearly reacted in different ways than the mesofauna. Comparable bimodal patterns are known from the benthic biomass distributions for coastal sediments [32].

### Biomass Fluxes

The relative energetic contribution to all consuming invertebrates has been computed. Energy use in local freshwater communities is reported as either independent of body size or decreasing with increasing body size [27]. As the energy acquisition is supposed to scale with body mass, the pattern of the resulting biomass flux as a fraction of the total flux in each food web is supposed to scale with the investigated body-size range. We speculate on possible mechanisms that may underlie a similar correlation between the biomass fluxes for the microfauna and the mesofauna and the soil pore structure. Within all these 135 investigated sites on Pleistocene sand, differences in the soil structure were small: soil particles were sand grains (90.62%±3.17 SD) and clay particles (2.88%±1.84 SD). If each soil particle is viewed as an island with a specific texture and nutrient content, different regions and resources within the soil come in contact through moisture films and can be reached by nematodes, as suggested by the faunal biomass for lower log(*M*)-values, like those binned between −0.6 and −0.8 (Figure 3). One lumped generalized linear model (hereafter, GLM) that assigned arable fields and forests to one category (ungrazed, unmanured), and all other sites to another category (grazed, manured), explained 54.6% of the variation on log total faunal biomass flux (GLM χ^2^ = 14.63, *p* = 0.0001).

This mechanism contributes to explain this striking correlation between the log total faunal biomass flux and both the cattle manure and the management regime (if any). As in our previous study on 68 of these 135 webs [5], [6], [20], nematodes were grazing most intensely on bacteria under manured conditions and greater livestock pressure, which implies that soil nematodes respond fast to fertilization-induced microbial pulses and strongly enhance energy and mass fluxes. In Figure 3, the binned biomass shows a remarkable increase within the 0.5 class (all the log(*M*)-values between 10^0.4^ and 10^0.6^, ranging from 2.51 to 3.98 µg dry adult weight). Most of these populations are fungivore oribatids which handle different resources than bacterivore nematodes. Possibly, these soil invertebrates are relevant “energy transfer agents”.

Human management variables (percent covered by grasses *vs.* cattle manure; Table 1) together explained significant additional variation in the soil community variables beyond: 91% *vs.* 86% (log total flux) and 68% *vs.* 56% (log total faunal biomass), *p*<0.0001 for both (percent decreases in unexplained variation, measured by 1−*R* ^2^, were 34% and 27% respectively). Seen the necessarily positive correlation between the occurrence of livestock and the extent of grass-covered open canopies, this strong covariance (degrees of freedom increased by 10 when these management variables were included) has to be expected. Management regime (mainly addition of P) thus substantially influenced the faunal community. The biomass flux differed significantly within the grazed category and between the categories (in both ANOVAs *p*<0.0001). The management regime within the grazed category is also correlated with a decreasing abundance of smaller invertebrates in relation to larger arthropods and enchytraeids (ANOVA *p*<0.0001 and GLM χ^2^ = 21.34, *p*<0.0001).

The synergy between soil structure and numerical abundance of larger organisms plays a key-role for the entire soil community food web. Especially the nutrient cycling in manured areas seems to be controlled by non-parasitic nematodes in the smallest log(*M*) bins [5], [6]. In addition, soil nematodes act as resource for many other invertebrates. From this perspective, the study of the relative distribution of total biomass fluxes in a food web seems to provide a fine tool to identify key invertebrates which play a specific role in ecosystem functioning due to their body size trait.

### Trophic Links

The density of the possible trophic links (Table 2) across a soil community is closely related to our six types of land use (ANOVA *p*<0.0001), as larger organisms, if present, tend to be more generalists: the higher the number of large-sized taxa the higher the number of possible trophic links. Our forest webs displayed the highest number of the species' total links (1737±460 SD); conventional farms and arable fields displayed the lowest amount (679±232 SD and 744±455 SD, respectively). Arable fields and dairy farms do this by limiting movements and access of large-sized animal groups, to all or part of their bacterial prey, as well as by supplying living space acting as refuges from predators for other animal groups or life stages (like the passive nematode stage Dauerlarvae [21]). The decrease of large-sized organisms in agroecosystems can be partially explained by land machineries and cattle trampling, and it confirms previous plate studies on bulk density by Yeates et al. [33].

For all 135 sites, the average±standard deviation of the number of trophic links was 993±517 (928±385 in the grazed agroecosystems and 1087±657 in the ungrazed agroecosystems). Averages of taxa (mostly genera) and trophic connections per agroecosystem were provided in Table 2. The average of the site-specific medians of the lengths of all the trophic links was 2.01±0.33 orders of magnitude, very close to the median of fields (Table 3). The larger the numerical abundance of the resource, the longer becomes the length of the trophic link for any given group of consumer numerical abundance [11], [26]. The median of the lengths of the trophic links in forests was by far the lowest, being only 1.43±0.12 orders of magnitude (Table 3). This implies that for the average link from one resource to its consumer, the ratio of the mean body mass of consumer to resource times the ratio of the numerical abundance of resource to consumer was about one hundred (10^1.43^–10^2.27^), assuming that the consuming species population had lower numerical abundance and larger mean body mass than its resource. Both the averages as the medians of the lengths of the trophic links between invertebrates were positively correlated with the total soil phosphorus availability (Pearson's Correlation Coefficients equal to 0.244, *p* = 0.003 and 0.942, *p*<0.0001, respectively), although the numbers of the trophic links themselves were negatively correlated with soil P (Pearson's Correlation equal to −0.320, *p*<0.0001).

**Table 3:** Topology of 135 real soil food webs.

	5^th^ percentile link length	median link length	95^th^ percentile link length	5^th^ percentile link slope	median link slope	95^th^ percentile link slope
organic grasslands	0.47±0.09	2.15±0.23	4.49±0.27	−2.88±0.51	−0.49±0.07	2.61±0.73
conventional farms	0.50±0.09	2.27±0.25	4.76±1.03	−3.26±0.70	−0.53±0.11	2.56±0.62
semi-intensive farms	0.49±0.08	2.14±0.29	4.52±1.09	−3.91±1.02	−0.57±0.08	2.84±0.87
intensive farms	0.43±0.06	1.94±0.24	4.26±0.31	−3.64±0.73	−0.45±0.09	3.45±0.88
forests	0.36±0.04	1.43±0.12	3.14±0.28	−5.41±1.27	−0.37±0.14	4.36±1.21
fields	0.42±0.07	2.03±0.18	4.46±0.63	−3.42±0.96	−0.44±0.11	3.01±0.68

The trophic link length difference between all kinds of agroecosystems was not significant at the 5% level according to a one-way ANOVA. Forests exhibited much shorter trophic links and a much higher variance in the slopes of the trophic links than the other agroecosystems. More explanations in the text.

The slope of each trophic link indicates the *biomass* ratio of the two coupled taxa. An isometric slope between *c* and *r* exactly equal to −1 implies that each unit of available resource biomass *B*_r_ supports a constant consumer biomass *B*_c_ (extensive literature review in [21]). A mass-abundance regression slope of −1 means thus that the consumer's biomass *N_c_×M_c_*, or, if log-scaled, log(*N*_c_)+log(*M*_c_), equals the resource biomass *N_r_×M_r_*, or log(*N*_r_)+log(*M*_r_), respectively. About 57% of the nematofauna graze on bacterial cells [11]. Taking again these bacterial-feeding invertebrates into account, we plotted them in a (*M*,*N*) plane with double logarithmical scale and body-mass averages M̅ as predictors of numerical abundances. A steeper slope (i.e., more negative than −1) indicates now that the microbial grazer *c* has smaller biomass than the bacterial resource *r*, seen that the microbial grazer is less abundant (but heavier) than the bacteria. The median slopes of our trophic links was −0.42±0.12 SD in the 55 ungrazed agroecosystems and −0.49±0.10 SD in the 80 grazed agroecosystems (Table 3), far away from the median slope of −1.03 in pelagic ecosystems [26], [34].

The most remarkable difference is shown by the average of the slopes of the trophic links occurring in our forested sites, where the resulting links tend to be flat (mean slope −0.06±3.74 SD due to dominant horizontal pairs with M̅_c_≈M̅_r_). This could at least point to a dominance of omnivory in forested sites in comparison to the other freshwater and terrestrial ecosystems. A comparison between forests and other ecosystems (Table 3) show how heterogeneous (possibly delayed) the belowground response can be to aboveground management. Bengtsson et al. [35] showed that in a “donor system” like detrital soil food webs, changes in the numerical abundance of organisms after harvesting (here, fields) were consistently dependent on the trophic position within the food web, whereas mobile collembolans and enchytraeids were enhanced by soil nutrient quality [21]. The situation with the microbial community is different, but also the soil microflora is known to be highly sensitive to environmental quality [36]–[38] and the microbial trophic links contribute significantly to the medians of trophic link lengths and trophic link slopes. Populations of small invertebrates vary more rapidly than those of larger invertebrates. Moreover, smaller, faster-growing nematodes have higher metabolic rates than larger, slower-growing arthropods.

### Ecological Implications

To date, very few studies include data from the entire belowground community size spectrum and a remarkable bias against small organisms still occurs [39]. Therefore the functional response of belowground soil communities to aboveground processes is not well understood yet. Being any food web linked to arbitrary spatial definitions and sampling techniques, the responses of soil invertebrates (typically restricted to microhabitat patches) and their functional traits have to be monitored carefully to evaluate the real implications for ecosystem services.

There have been only some studies describing the actual impact of grazing cattle and application of manure on the abundance and biodiversity of soil faunal communities. Effects on the litter and soil fauna related to increasing stocking intensity have been recognized –among others– by King et al. [40] in the springtails' community, by Kay et al. [41] in the mite assemblages and by Mulder et al. [5], [6] in the nematofauna. These authors found that the numerical abundance of microbivores (for mites, in particular those belonging to the families Nanorchestidae, Tarsonemidae and Tydeidae; for soil nematodes, both the active bacterial-grazers as the hyphal-feeders) declined with livestock intensity and were much lower in grazed pastures.

Habitat-induced biomass clumps in the belowground distribution of invertebrates' body-sizes are evident, in contrast to the total amounts of possible trophic interactions which are positively correlated with the belowground faunal biodiversity (*p*<0.0001) and are unaffected by soil heterogeneity (*p* = 0.16). The lower faunal biomass in ungrazed locations implies thus slower energy flux and lesser matter turnover than in grazed locations. Therefore, management-induced changes in the body-size distribution (with the previously discussed shifts between nematodes and microarthropods) may compensate influences on the consumption per mass unit.

We believe that our structural approach contributes towards an extensive comparison of ecosystems and enables the recognition of sensitive non-target body-size classes. We have statistically modelled this variation in the soil faunal biomass distribution and biomass flux; showed that occurrence of livestock is a reliable allometric predictor; and assessed that cattle manure enhances lower body-size clumps in the faunal biomass distribution. According to us, these results might provide new empirical evidence that body size matters also in terrestrial ecosystems.

## Supporting Information

Table S1 Soil Taxonomical Inventory and Dominant Feeding-Strategies. Each taxon, regardless of the taxonomic resolution, is defined by a 5-digit code. The first digit (1 up to 9) provides information on the dominant feeding strategy as provided in Table S1: 1 = Plant-feeder, 2 = Fungivore, 3 = Bacterivore, 4 = Substrate ingestion, 5 = Predator of nematodes, 6 = Predator of arthropods, 7 = General predator (predator of nematodes and of microarthropods, but no parasitizing life stage), 8 = Omnivore (generalist, predator, plant-feeder and/or fungivore, possibly parasite), and 9 = Parasite (hosts are mites or nematodes; no passive dispersal of deutonymphs by phoresy). The second digit (0 up to 5) provides cladistic information: 0 = Bacterial cells (no taxonomical definition possible, all species lumped together), 1 = Nematoda, 2 = Acarina , 3 = Insecta (Collembola, Protura, Diplura, Myriapoda, Pauropoda, and Symphyla), 4 = Enchytraeidae, and 5 = Lumbricidae. The last three digits define the occurring taxon. The additional references are provided as well.
(0.09 MB PDF)
