# Supplementary material for: Aboveground Herbivory Shapes the Biomass Distribution and Flux of Soil Invertebrates
Source: PLoS One. 2008 Oct 31;3(10):e3573. doi: 10.1371/journal.pone.0003573 (PMC2570614; doi:10.1371/journal.pone.0003573)
Supplement: Table S1 — Soil Taxonomical Inventory and Dominant Feeding-Strategies. Each taxon, regardless of the taxonomic resolution, is defined by a 5-digit code. The first digit (1 up to 9) provides information on the dominant feeding strategy as provided in Table S1: 1 = Plant-feeder, 2 = Fungivore, 3 = Bacterivore, 4 = Substrate ingestion, 5 = Predator of nematodes, 6 = Predator of arthropods, 7 = General predator (predator of nematodes and of microarthropods, but no parasitizing life stage), 8 = Omnivore (generalist, predator, plant-feeder and/or fungivore, possibly parasite), and 9 = Parasite (hosts are mites or nematodes; no passive dispersal of deutonymphs by phoresy). The second digit (0 up to 5) provides cladistic information: 0 = Bacterial cells (no taxonomical definition possible, all species lumped together), 1 = Nematoda, 2 = Acarina , 3 = Insecta (Collembola, Protura, Diplura, Myriapoda, Pauropoda, and Symphyla), 4 = Enchytraeidae, and 5 = Lumbricidae. The last three digits define the occurring taxon. The additional references are provided as well. (0.09 MB PDF) [file pone.0003573.s001.pdf]

Table S1 – CODE OF THE TROPHIC SPECIES (5 NUMBERS, 378 TAXA)

1st NUMBER (1 up to 9) PROVIDE INFORMATION ON THE **FEEDING STRATEGY**

1 = Plant-feeder
2 = Fungivore
3 = Bacterivore
4 = Substrate ingestion
5 = Predator of nematodes
6 = Predator of arthropods
7 = General predator (predator of nematodes and of arthropods, but no parasitizing life stage)
8 = Omnivore (generalist, predator, plant-feeder and/or fungivore, possibly parasite)
9 = Parasite (hosts are mites or nematodes; no passive dispersal of deutonymphs by phoresy)

2nd NUMBER (0 up to 5) PROVIDES INFORMATION ON THE **TAXOCENES** and BODY-WIDTH RANGE OF EUKARYOTES IN RELATION TO THE SOIL STRUCTURE: a. *microfauna*: decomposers, inhabit water films; b. *mesofauna*: predators and microbial grazers, inhabit air-filled pore spaces, use existing pore structures not altering pore structure; c. *macrofauna*: create their own spaces through burrowing activities, thus changing pore structure

PROKARYOTES

MICROFLORA (MICROBES)
0 **Bacteria** (no taxonomical definition possible, all species lumped together, code "0000")

EUKARYOTES

Class Nematoda

MICROFAUNA
1 **Nematoda** (169 taxa)

Phylum Arthropoda
MESOFAUNA
2 *Acarina* ("Mites", 146 taxa). Four suborders:
Oribatei ("K" strategists),
Prostigmata (*r*-strategists),
Mesostigmata, and
Astigmata
Generally, very little is known about the niches or ecological requirements of most soil mite species

3 *Insecta* (Subclass Apterygota)
Collembola – springtails, wingless hexapodous microarthropods
Protura – closely related to collembolans, minute, slender and wingless soil microarthropods
Diplura – japygids are predators on small arthropods, nematodes and enchytraeids or saprophagous feeders
Myriapoda
Pauropoda – blind, sense organs include peculiar 2-branched antennae, 3 long flagella
Symphyla – resemble tiny centipedes

Class Oligochaeta

MESOFAUNA
4 Family Enchytraeidae ("potworms", 8 genera), anatomically similar to tiny earthworms

MACROFAUNA
5 Family Lumbricidae ("Earthworms", 9 genera)

Achromadora	21001
Acrobeles	31002
Acrobeloides	31003
Acrobelophis	31004
Acrolobus	31005
Aglenchus	11006
Alaimidae	31007
Alaimus	31008
Allodorylaimus	81009
Amphidelus	31010
Amplimerlinius	11011
Anaplectus	31012
Anatonchus	51013
Anguinidae	11014
Aphelenchidae	21015
Aphelenchoides	21016
Aphelenchoididae	21017
Aphelenchus	21018
Aporcelaimellus	81019
Aporcelaimidae	81020
Aporcelaimus	81021
Basiria	11022
Bastiania	31023
Bastianiidae	31024
Bitylenchus	11025
Boleodorus	11026
Bunonema	31027
Bunonematidae	31028
Bursilla	31029
Butlerius	31030
Cephalenchus	31031
Cephalobidae	31032
Cephalobus	31033
Cervidellus	31034
Chiloplacus	31035
Chromadoridae	31036
Chronogaster	31037
Clarkus	51038
Coarctadera	31039
Coomansus	51040
Coslenchus	11041
Criconematidae	11042
Cruznema	31043
Cuticularia	31044
Cylindrolaimus	31045
Dauerlarvae stage	41046
Deladenus	91047
Diphtherophora	21048
Diplogaster	81049
Diplogasteridae	81050
Diploscapter	31051
Discolaimus	51052
Ditylenchus	21053
Dolichodoridae	11054
Dolichorhabditis	31055
Dolichorhynchus	11056
Dorydorella	81057
Dorylaimoidea	81058
Dorylaimoides	81059
Dorylaimus	81060
Drilocephalobus	31061
Ecphyadophora	11062
Ecumenicus	81063
Epidorylaimus	81064
Eucephalobus	31065
Eudorylaimus	81066
Eumonhystera	31067
Euteratocephalus	31068
Fictor	31069
Filenchus	11070
Geocenamus	11071
Geomonhystera	31072
Gracilacus	11073
Helicotylenchus	11074
Hemicycliophora	11075
Heterocephalobus	31076
Heterodera	11077
Heteroderidae	11078
Heterorhabditis	91079
Hoplolaimidae	11080
Labronema	81081
Laimydorus	81082
Lelenchus	11083
Leptonchidae	21084
Longidorella	11085
Longidoridae	11086
Longidorus	11087
Macroposthonia	11088
Macrotrophurus	11089
Malenchus	11090
Meloidogyne	11091
Merlinius	11092
Mesodorylaimus	81093
Mesorhabditis	31094
Metateratocephalus	31095
Microdorylaimus	81096
Microlaimidae	31097
Microlaimus	31098
Monhystera	31099
Monhysteridae	31100
Monhystrella	31101
Mononchidae	51102
Mononchoides	51103
Mononchus	51104
Mydonomidae	81105
Mylonchulus	51106
Nagelus	11107
Neodiplogasteridae	81108
Neopsilenchus	11109
Neothada	11110
Nordiidae	81111
Nothotylenchus	21112
Odontolaimus	31113
Panagrobelus	31114
Panagrolaimidae	31115
Panagrolaimus	31116
Paramphidelus	31117
Paraphanolaimus	31118
Paratrichodorus	11119
Paratrophurus	11120
Paratylenchidae	11121
Paratylenchus	11122
Pareudiplogaster	31123
Pellioditis	31124
Pelodera	31125
Plectidae	31126
Plectus	31127
Pratylenchidae	11128
Pratylenchus	11129
Prionchulus	51130
Prismatolaimus	31131
Pristionchus	31132
Prodesmodora	31133
Prodorylaimus	81134
Protorhabditis	31135
Pseudhalenchus	21136
Psilenchidae	11137
Psilenchus	11138
Pungentus	81139
Qudsianematidae	81140
Quinisulcius	11141
Rhabditidae	31142
Rhabditis	31143
Rotylenchus	11144
Scutylenchus	11145
Seinura	51146
Steinernematidae	91147
Teratocephalidae	31148
Teratocephalus	31149
Thonus	51150
Thornematidae	81151
Thornia	81152
Torumanawa	81153
Trichodoridae	11154
Trichodorus	11155
Tripyla	51156
Tripylidae	51157
Trischistoma	51158
Trophurus	11159
Tylenchidae	21160
Tylencholaimellus	21161
Tylencholaimus	21162
Tylenchorhynchus	11163
Tylenchus	21164
Tylocephalus	31165
Tylolaimophorus	21166
Tylopharynx	31167
Wilsonema	31168
Xiphinema	11169

NEMATODA, 2nd NUMBER = 1, THE 3rd-5th NUMBERS ARE GENERA/FAMILIES

FEEDING STRATEGIES:

1 = Plant-feeder, 2 = Fungivore, 3 = Bacterivore, 4 = Substrate ingestion (only dauerlarvae), 5 = Predators of nematodes, 8 = Omnivore (1+2+5), 9 = Parasite

Achipteria	12001
Adamaeus	22002
Adoristes	22003
Alliphis	52004
Amblyseius	62005
Anystidae	72006
Arctoseius	72007
Asca	72008
Astegistes	22009
Astigmata	82010
Atropacarus	12011
Balaustium	92012
Banksinoma	22013
Bdella	62014
Bdellidae	62015
Belbidae	22016
Berniniella	22017
Blattisocius	62018
Brachychthoniidae	22019
Brachychthonius	22020
Camisia	12021
Cepheus	22022
Ceratoppia	22023
Ceratozetes	82024
Chamobates	22025
Cheiroseius (=Sejus)	72026
Cilliba	82027
Coccotydeus	12028
Cosmochthonius	22029
Cyrtolaelaps	72030
Damaeidae (*sensu lato*)	22031
Damaeobelba	22032
Damaeus	22033
Dendrolaelaps	72034
Dendroseius	72035
Diapterobates	22036
Dissorhina	22037
Diversipes	22038
Eniochthonius	22039
Epicriopsis	22040
Ereynetidae	92041
Eriophyidae	12042
Erythraeidae	82043
Eulohmannia	22044
Eupelops	32045
Eupodes	82046
Eupodidae	82047
Euzetes	22048
Galumna	12049
Gamasodes	72050
Gamasolaelaps	72051
Geholaspis	62052
Hemileius	22053
Hermannia	22054
Histiostoma	32055
Holoparasitus	72056
Humerobates	82057
Hypoaspis	72058
Hypochthonius	22059
Imparipes	22060
Isozercon	72061
Johnstonianidae	92062
Labidostomma	72063
Lasioseius	72064
Lauroppia	22065
Ledermuelleria	12066
Leioseius	72067
Liebstadia	22068
Linopodes	22069
Liochthonius	22070
Lysigamasus	72071
Macrocheles	72072
Macrochelidae	72073
Medioppia	22074
Mesostigmata (juven.)	82075
Metabelba	22076
Microppia	22077
Microtritia	12078
Microtydeus	22079
Minunthozetes	22080
Nanhermannia	22081
Nanorchestes	12082
Nenteria	82083
Neojordensia	72084
Nothrus	12085
Odontocepheus	22086
Oppiella	22087
Oppiidae	22088
Oribatella	22089
Oribatida (juveniles)	82090
Oribatula	12091
Pachygnatidae	12092
Pachylaelaps	72093
Pachyseius	72094
Palaeacarus	22095
Parasitus	72096
Parazercon	72097
Penthaleus	12098
Penthalodidae	12099
Pergamasus	72100
Phauloppia	22101
Phthiracarus	12102
Phytoseiidae	62103
Platynothrus	12104
Prostigmata	92105
Protodinychus	82106
Prozercon	72107
Pseudoparasitus	92108
Punctoribates	22109
Pyemotes	92110
Pyemotidae	92111
Pygmephorus	22112
Quadroppia	22113
Ramusella	22114
Rhagidia	72115
Rhagidiidae	72116
Rhizoglyphus	12117
Rhodacarellus	72118
Rhodacaridae	72119
Rhodacarus	72120
Rhysotritia	22121
Scheloribates	82122
Schwiebea	22123
Scutacaridae	82124
Scutacarus	82125
Sellnickochthonius	22126
Siteroptes	22127
Spatiodamaeus	22128
Speleorchestes	22129
Steganacarus	12130
Stigmaeidae	82131
Suctobelbella	22132
Tarsonemidae	92133
Tarsonemus	82134
Tectocepheus	22135
Trachytes	22136
Trichoribates	22137
Trombidiidae	92138
Tydeidae	12139
Tyrophagus	22140
Uroobovella	72141
Uropoda	72142
Uroseius	72143
Veigaia	72144
Zercon	72145
Zercoseius	72146

ACARINA (=MITES), THE 2nd NUMBER = 2, 3rd-5th NUMBERS ARE MOSTLY GENERA

THE DOMINANT FEEDING STRATEGIES AS DERIVED FROM FEEDING GUILDS:

1 = Plant-feeder (macrophytophages+panphytophages)
2 = Fungivore (microphytophages)
3 = Bacterivore
4 = Substrate ingestion (does not occur in micro-arthropods)
5 = Predator of nematodes
6 = Predator of arthropods
7 = General predator (5+6, no 9)
8 = Omnivore (7+1+2, poss. +9)
9 = Parasite (hosts are mites or nematodes; no passive dispersal of deutonymphs by phoresy)

INSECTS AND OTHERS

Allacma	13001
Anurida	23002
Brachystomella	23003
Ceratophysella	23004
Dicyrtoma	13005
DIPLURA	83006
Entomobrya	13007
Entomobryidae	13008
Folsomia	23009
Friesea	23010
Heteromurus	23011
Hypogastrura	23012
Isotoma	23013
Isotomiella	23014
Isotomurus	23015
Lepidocyrtus	23016
Megalothorax	23017
Mesaphorura	23018
Neanura	23019
Neelides	23020
Onychiurus	23021
Orchesella	23022
Paratullbergia	23023
Parisotoma	23024
PAUROPODA	23025
Proisotoma	23026
Protaphorura	23027
PROTURA	23028
Pseudachorutes	23029
Pseudisotoma	23030
Pseudosinella	23031
Sminthuridae	13032
Sminthurides	13033
Sminthurinus	13034
Sminthurus	13035
Sphaeridia	13036
Stenaphorurella	23037
SYMPHYLA	13038
Tomocerus	23039
Vertagopus	23040
Willemia	23041

THE SECOND NUMBER (“3”) IS ONLY FOR COLLEMBOLA, DIPLURA, PAUROPODA, PROTURA AND SYMPHYLA

FIRST NUMBER REFLECTS THE FEEDING-STRATEGY OF THESE ARTHROPODS: 1=plant-feeder, 2=fungivore, or 8=omnivore (1+2+7). OTHER FOUR NUMBERS PROVIDE TAXONOMIC INFORMATION

OLIGOCHAETA

Juveniles (undiff/indet)	44000
Achaeta	24001
Buchholzia	44002
Cognettia	24003
Enchytraeus	34004
Enchytronia	34005
Fridericia	24006
Hemienchytraeus	34007
Hemifridericia	24008
Henlea	44009
Marionina	44010
Mesenchytraeus	34011
Allobophora	45012
Aporrectodea	45013
Dendrobaena	45014
Dendrodrilus	45015
Eisenia	45016
Eiseniella	45017
Lumbricus	45018
Octolasion	45019
Satchellius	45020

OLIGOCHAETA (=WORMS) ENCHYTRAEIDAE (2nd NUMBER = 4) AND LUMBRICIDAE (2nd NUMBER =5), THE 3rd-5th NUMBERS ARE GENERA OR LIFE-STAGES

FEEDING STRATEGIES:

2 = Fungivore
3 = Bacterivore
4 = Substrate ingestion

Please note: the feeding strategy **9 (= Parasite)** never occurs. These worms are <u>not</u> leeches.

BACTERIAL CELLS HAVE NOT BEEN RECOGNIZED AT TAXONOMICAL LEVEL, AND ARE ENTERED IN THE DATASET WITH THE **CODE 40000** (MORPHOSPECIES).

References used to establish trophic relationships

Agrawal, A. A., Kobayashi, C. & Thaler, J. S. 1999 Influence of prey availability and induced host-plant resistance on omnivory by western flower thrips. *Ecology* 80:518-523.

Axelsson, B., Lohm, U. & Persson, T. 1984 Enchytraeids, lumbricids and soil arthropods in a northern deciduous woodland – a quantitative study. *Holarctic Ecology* 7:91-103.

Bardgett, R. D. & Chan, K. F. 1999 Experimental evidence that soil fauna enhance nutrient mineralization and plant nutrient uptake in montane grassland ecosystems. *Soil Biology and Biochemistry* 31:1007-1014.

Bardgett, R. D. & Cook, R. 1998 Functional aspects of soil animal diversity in agricultural grasslands. *Applied Soil Ecology* 10:263-276.

Bardgett, R. D., Cook, R., Yeates, G. W. & Denton, C. S. 1999 The influence of nematodes on below-ground processes in grassland ecosystems. *Plant and Soil* 212:23-33.

Bardgett, R. D., Keiller, S., Cook, R. & Gilburn, A. S. 1998 Dynamic interactions between soil animals and microorganisms in upland grassland soils amended with sheep dung: a microcosm experiment. *Soil Biology and Biochemistry* 30:531-539

Bardgett, R. D., Wardle, D. A. & Yeates, G. W. 1998 Linking above-ground and below-ground interactions: how plant responses to foliar herbivory influence soil organisms. *Soil Biology and Biochemistry* 30:1867-1878.

Berg, M. P., Stoffer, M. & Van den Heuvel, H. H. 2002 Feeding guilds in Collembola based on digestive enzymes. *Pedobiologia* 48: 589-601.

Chen, B. R. & Wise, D. H. 1997 Responses of forest-floor fungivores to experimental food enhancement. *Pedobiologia* 41:316-326.

Cross, E. A. & Moser, J. C. 1975 A new dimorphic species of *Pyemotes* and a key to previously-described forms (Acarina: Tarsonemoidea). *Annals of the Entomological Society of America* 68:723-732.

Cross, E. A., Moser, J. C. & Rack, G. 1981 Some new forms of *Pyemotes* (Acarina: Pyemotidae) from forest insects, with remarks on polymorphism. *International Journal of Acarology* 7:179-196.

De Ruiter, P. C., Van Veen, J. A., Moore, J. C., Brussaard, L. & Hunt, H. W. 1993 Calculation of nitrogen mineralisation in soil food webs. *Plant and Soil* 157:263-273.

Díaz, A., Okabe, K., Eckenrode, C. J., Villani, M. G. & O'Connor, B. M. 2000 Biology, ecology, and management of the bulb mites of the genus *Rhizoglyphus* (Acari: Acaridae). *Experimental and Applied Acarology* 24:85-113.

Dindal, D. L. 1990 *Soil biology guide*. John Wiley & Sons: New York.

Enkegaard, A., Sardar, M. A. & Brødsgaard, H. F. 1997 The predatory mite *Hypoaspis miles*: biological and demographic characteristics on two prey species, the mushroom sciarid fly, *Lycoriella solani*, and the mould mite, *Tyrophagus putrescentiae*. *Entomologia Experimentalis et Applicata* 82:135-146.

Gange, A. C. 1993 Translocation of mycorrhizal fungi by earthworms during early plant succession. *Soil Biology and Biochemistry* 25:1021-1026

Gange, A. C. 2000*a* Species-specific responses of a root- and shoot-feeding insect to arbuscular mycorrhizal colonization of its host plant. *New Phytologist* 150:611-618

Gange, A. C. 2000*b* Arbuscular mycorrhizal fungi, Collembola and plant growth. *Trends in Ecology and Evolution* 15:369-372.

Gange, A. C. & Brown, V. K. 2002 Soil food web components affect plant community structure during early succession. *Ecological Research* 17:217-227.

Hunt, H. W., Coleman, D. C., Ingham, E. R., Ingham, R. E., Elliot, E. T., Moore, J. C., Rose, S. L., Reid, C. P. P. & Morley C. R. 1987 The detrital food web in a shortgrass prairie. *Biology and Fertility of Soils* 3:57-68.

Ingham, R. E., Trofymow, J. A., Ingham, E. R. & Coleman, D. C. 1985 Interactions of bacteria, fungi, and their nematode grazers: Effects on nutrient cycling and plant growth. *Ecological Monographs* 55:119-140.

Janssen, A., Pallini, A., Venzon, M. & Sabelis, M. W. 1998 Behaviour and indirect interactions in food webs of plant-inhabiting arthropods. *Experimental and Applied Acarology* 22:497-521.

Kay, F. R., Sobhy, H. M. & Whitford, W. G. 1999 Soil microarthropods as indicators of exposure to environmental stress in Chihuahuan desert Rangelands. *Biology and Fertility of Soils* 28:121–128.

King, K. L., Hutchinson, K. J. & Greenslade, P. 1976 The effects of sheep numbers on associations of Collembola in sown pastures. *Journal of Applied Ecology* 13:731–739.

Klironomos, J. N. & Kendrick, B. 1995*a* Relationships among microarthropods, fungi, and their environment. *Plant and Soil* 170:183-197.

Klironomos, J. N. & Kendrick, B. 1995*b*. Stimulative effects of arthropods on endomycorrhizas of sugar maple in the presence of decaying litter. *Functional Ecology* 9:528-536

Klironomos, J. N. & Kendrick, W. B. 1996 Palatability of

microfungi to soil arthropods in relation to the functioning of arbuscular mycorrhizae. *Biology and Fertility of Soils* 21:43-52.

Laakso, J. & Setälä, H. 1999 Population- and ecosystem-level effects of predation on microbial-feeding nematodes. *Oecologia* 120:279-286.

Laakso, J., Setälä, H. & Palojärvi, A. 2000 Influence of decomposer food web structure and nitrogen availability on plant growth. *Plant and Soil* 225:153-165.

Liiri, M., Setälä, H., Haimi, J., Pennanen, T. & Fritze, H. 2002*a* Relationship between soil microarthropod species diversity and plant growth does not change when the system is disturbed. *Oikos* 96:137-149.

Liiri, M., Setälä, H., Haimi, J., Pennanen, T. & Fritze, H. 2002*b* Soil processes are not influenced by the functional complexity of soil decomposer food webs under disturbance. *Soil Biology and Biochemistry* 34:1009-1020.

Marshall, V. G., Reeves, R. M. & Norton, R. A. 1987 Catalogue of the Oribatida (Acari) of continental United States and Canada. *Memoirs of the Entomological Society of Canada* 139:1-623.

Moore, J. C., Walter, D. E. & Hunt, H. W. 1988 Arthropod regulation of micro- and mesobiota in below-ground detrital food web. *Annual Reviews of Entomology* 33:419-439.

Mulder, Ch., Van Wijnen, H. J. & Van Wezel, A. P. 2005*a* Numerical abundance and biodiversity of below-ground taxocenes along a pH gradient across the Netherlands. *Journal of Biogeography* 32:1775-1790.

Mulder, Ch., Cohen, J. E., Setälä, H., Bloem, J. & Breure, A. M. 2005*b* Bacterial traits, organism mass, and numerical abundance in the detrital soil food web of Dutch agricultural grasslands. *Ecology Letters* 8:80-90 (Supplementary Online Material free available at http://www.blackwellpublishing.com/products/journals/suppmat/ELE/ELE704/ELE704sm.htm).

Mulder, Ch., Dijkstra, J.B. & Setälä, H. 2005*c* Nonparasitic Nematoda provide evidence for a linear response of functionally important soil biota to increasing livestock density. *Naturwissenschaften* 92:314-318.

Mulder, Ch., Schouten, A. J., Hund-Rinke, K. & Breure, A. M. 2005*d* The use of nematodes in ecological soil classification and assessment concepts. *Ecotoxicology and Environmental Safety (Environmental Research Section B)* 62: 278-289.

Okada, H., Tsukiboshi, T. & Kadota, I. 2002 Mycetophagy in *Filenchus misellus* (Andrássy, 1958) Lownsbery & Lownsbery, 1985 (Nematoda: Tylenchidae) with notes on its morphology. *Nematology* 4:795-801.

Patt, J. M., Wainright, S. C., Hamilton, G. C., Whittinghill, D., Bosley, K., Dietrick, J. & Lashomb, J. H. 2003 Assimilation of carbon and nitrogen from pollen and nectar by a predaceous larva and its effects on growth and development. *Ecological Entomology* 28:717-728.

Perez-Moreno, J. & Read, D. J. 2001 Nutrient transfer from soil nematodes to plants: a direct pathway provided by the mycorrhizal mycelial network. *Plant, Cell and Environment* 24:1219-1226.

Petersen, H. 1981 The respiratory metabolism of Collembola species from a Danish beech wood. *Oikos* 37:273-286.

Petersen, H. 1995*a* Temporal and spatial dynamics of soil Collembola during secondary succession in Danish heathland. *Acta Zoologica Fennica* 196:190-194.

Petersen, H. 1995*b* Energy flow and trophic relations in soil communities: state of knowledge two decades after the International biological Programme. In: Edwards, C. A., Abe, T. & Striganova, B. R. (eds.) *Structure and Function of Soil Communities*. Kyoto University Press, p. 111-130.

Petersen, H. 2000 Collembola populations in an organic crop rotation: population dynamics and metabolism

after conversion from clover-grass ley to spring barley. *Pedobiologia* 44:502-515.

Petersen, H. 2002 Effects of non-inverting deep tillage vs. conventional ploughing on collembolan populations in an organic wheat field. *European Journal of Soil Biology* 38:177-180.

Petersen, H. & Gjelstrup, P. 1998 Effects of the insecticide dimethoate on the behaviour of *Folsomia fimetaria* (L.) (Collembola: Isotomidae). *Applied Soil Ecology* 9:389-392.

Petersen, H. & Luxton, M. 1982 A comparative analysis of soil fauna populations and their role in decomposition process. *Oikos* 39:288-388.

Petersen, H., Jucevica, E. & Gjelstrup, P. 2004 Long-term changes in collembolan communities in grazed and non-grazed abandoned arable fields in Denmark. *Pedobiologia* 48:559-573.

Phillipson, J., Abel, R., Steel, J. & Woodell, S. R. J. 1977 Nematode numbers, biomass and respiratory metabolism in a beech woodland – Wytham Woods, Oxford. *Oecologia* 27:141-155.

Radwan, J. & Bogacz, I. 2000 Comparison of life-history traits of the two male morphs of the bulb mite, *Rhizoglyphus robini*. *Experimental and Applied Acarology* 24:115-121.

Rantalainen, M.-L., Fritze, H., Haimi, J., Kiikkilä, O., Pennanen, T. & Setälä, H. 2004 Do enchytraeid worms and habitat corridors facilitate the colonization of habitat patches by soil microbes? *Biology and Fertility of Soils* 39:200-208.

Scheu, S. & Falca, M. 2000 The soil food web of two beech forests (*Fagus sylvatica*) of contrasting humus type: stable isotope analysis of a macro- and a mesofauna-dominated community. *Oecologia*, 123:285-296.

Scheu, S. & Folger, M. 2004 Single and mixed diets in Collembola: effects on reproduction and stable isotope fractionation. *Functional Ecology* 18:94-102.

Scheu, S. & Setälä, H. 2002 Multitrophic interactions in decomposer food-webs. In: Tscharntke, T. & Hawkins B. A. (eds.) *Multitrophic Level Interactions*. Cambridge University Press: Cambridge, p. 223-264.

Scheu, S. & Simmerling, F. 2004 Growth and reproduction of fungal feeding Collembola as affected by fungal species, melanin and mixed diets. *Oecologia* 139:347-353.

Scheu, S., Theenhaus, A. & Jones, T. H. 1999 Links between the detrivore and the herbivore system: effects of earthworms and Collembola on plant growth and aphid development. *Oecologia* 119:541-551.

Schönholzer, F., Hahn, D. & Zeyer, J. 1999 Origins and fate of fungi and bacteria in the gut of *Lumbricus terrestris* L. studied by image analysis. *FEMS Microbiology Ecology* 28:235-248.

Setälä, H. 1995 Growth of birch and pine seedlings in relation to grazing by soil fauna on ectomycorrhizal fungi. *Ecology* 76:1844-1851.

Setälä, H. 2002 Sensitivity of ecosystem functioning to changes in trophic structure, functional group composition and species diversity in belowground food webs. *Ecological Research* 17:207-215.

Setälä, H., Tyynismaa, M., Martikainen, E. & Huhta, V. 1991 Mineralization of C, N and P in relation to decomposer community structure in coniferous forest soil. *Pedobiologia* 35:285-296.

Setälä, H., Laakso, J., Mikola, J. & Huhta, V. 1998 Functional diversity of decomposer organisms in relation to primary production. *Applied Soil Ecology* 9:25-31.

Siemann, E., Tilman, D. & Haarstad, J. 1999 Abundance, diversity and body size: patterns from a grassland arthropod community. *Journal of Animal Ecology* 68:824-835

Siepel, H. 1994 Life-history tactics of soil microarthropods. *Biology and Fertility of Soils* 18:263-278.

Siepel, H. & De Ruiter-Dijkman, E. M. 1993 Feeding guilds of oribatid mites based on carbohydrase enzyme activities. *Soil*

Biology and Biochemistry 25:1491-1497.

Siepel, H. & Maaskamp, F. 1994 Mites of different feeding guilds affect decomposition of organic matter. *Soil Biology and Biochemistry* 26:1389–1394.

Small, R. W. 1987 A review of the prey of predatory soil nematodes. *Pedobiologia* 30:179-206.

Van Straalen, N. M. 1982 Demographic analysis of arthropod populations using a continuous stage-variable. *Journal of Animal Ecology* 51:769-783.

Van Straalen, N. M. 1985 Comparative demography of forest floor Collembola populations. *Oikos* 45:253-265.

Van Straalen, N. M. 1987 Turnover of accumulating substances in populations with weight-structure. *Ecological Modelling* 36:195-209.

Van Straalen, N. M. 1989 Production and biomass turnover in two populations of forest floor Collembola. *Netherlands Journal of Zoology* 39:156-168.

Van Straalen, N. M. & Verhoef, H. A. 1997 The development of a bioindicator system for soil acidity based on arthropod pH preferences. *Journal of Applied Ecology* 34:217-232.

Vegter, J. J. 1983 Food and habitat specialization in coexisting springtails (Collembola, Entomobryidae). *Pedobiologia* 25:253-262.

Vegter, J. J. 1987 Phenology and seasonal resource partitioning in forest floor Collembola. *Oikos* 48:175-185.

Vegter, J. J., De Bie, P. & Dop, H. 1988*a* Distributional ecology of forest floor Collembola (Entomobryidae) in the Netherlands. *Pedobiologia* 31:65-73.

Vegter, J. J., Joosse, E. N. G. & Ernsting, G. 1988*b* Community structure, distribution and population dynamics of Entomobryidae (Collembola). *Journal of Animal Ecology* 57:971-981.

Verhoef, H. A. 1995 Animal ecophysiology: cornerstone for soil ecosystem studies as exemplified by studies on arthropods. *Acta Zoologica Fennica* 196:176-182.

Vreeken Buijs, M. J., Geurs, M., De Ruiter, P. C. & Brussaard, L. 1997 The effects of bacterivorous mites and amoebae on mineralization in a detrital based below-ground food web; microcosm experiment and simulation of interactions. *Pedobiologia* 41:481-493.

Wardle, D. A., Yeates, G. W., Watson, R. N. & Nicholson, K. S. 1995 development of the decomposer food-web, trophic relationships, and ecosystem properties during a three year primary succession in sawdust. *Oikos* 73:155-166.

Wardle, D. A., Barker, G. M., Yeates, G. W., Bonner, K. I. & Ghani, A. 2001 Introduced browsing mammals in New Zealand natural forests: Aboveground and belowground consequences. *Ecological Monographs* 71:587-614.

Wolters, V. 1985 Resource allocation in *Tomocerus flavescens* (Insecta, Collembola): a study with C-14-labelled food. *Oecologia* 65:229-235.

Wolters, V. 1998 Long-term dynamics of a collembolan community. *Applied Soil Ecology* 9:221-227.

Yeates, G. W. 1979 Soil nematodes in terrestrial ecosystems. *Journal of Nematology* 11:213-229.

Yeates, G. W. 2003 Nematodes as soil indicators: functional and biodiversity aspects. *Biology and Fertility of Soils* 37:199-210.

Yeates, G. W., Bongers, T., De Goede, R. G. M., Freckman, D. W. & Georgieva, S. S. 1993 Feeding habits in soil nematode families and genera – an outline for soil ecologists. *Journal of Nematology* 25:315-331.

Yeates, G. W., Saggar, S., Denton, C. S. & Mercer, C. F. 1998 Impact of clover cyst nematode (*Heterodera trifolii*) infection on soil microbial activity in the rhizosphere of white clover (*Trifolium repens):* a pulse labelling experiment. *Nematologica* 44:81–90.
